# Sialyllactose preserves residual hearing after cochlear implantation

**DOI:** 10.1038/s41598-024-62344-0

**Published:** 2024-06-10

**Authors:** Min Young Lee, Seo-Kyung Jung, Jongmoon Jang, Hongsoo Choi, Yun-Hoon Choung, Jeong Hun Jang

**Affiliations:** 1 Department of Otolaryngology, Dankook University Hospital, Cheonan, Republic of Korea; 2 Department of Otorhinolaryngology, Ajou University School of Medicine, San 5, Wonchon-dong, Yeongtong-gu, Suwon, 443-721 Republic of Korea; 3 Department of Functional Ceramics, Korea Institute of Materials Science (KIMS), Changwon, Republic of Korea; 4 Department of Robotics Engineering, Daegu Gyeongbuk Institute of Science and Technology (DGIST), Daegu, Republic of Korea

**Keywords:** Hearing loss, Cochlear implant, Residual hearing, Anti-inflammatory drug, Drug development, Experimental models of disease, Medical research, Neurology, Neurological disorders

## Abstract

In individuals with hearing loss, protection of residual hearing is essential following cochlear implantation to facilitate acoustic and electric hearing. Hearing preservation requires slow insertion, atraumatic electrode and delivery of the optimal quantity of a pharmacological agent. Several studies have reported variable hearing outcomes with osmotic pump-mediated steroid delivery. New drugs, such as sialyllactose (SL) which have anti-inflammatory effect in many body parts, can prevent tissue overgrowth. In the present study, the positive effects of the pharmacological agent SL against insults were evaluated in vitro using HEI-OC1 cells. An animal model to simulate the damage due to electrode insertion during cochlear implantation was used. SL was delivered using osmotic pumps to prevent loss of the residual hearing in this animal model. Hearing deterioration, tissue fibrosis and ossification were confirmed in this animal model. Increased gene expressions of inflammatory cytokines were identified in the cochleae following dummy electrode insertion. Following the administration of SL, insertion led to a decrease in hearing threshold shifts, tissue reactions, and inflammatory markers. These results emphasize the possible role of SL in hearing preservation and improve our understanding of the mechanism underlying hearing loss after cochlear implantation.

## Introduction

Sensorineural hearing loss is caused by various conditions, such as aging, acoustic overexposure, genetics, and ototoxic drugs. Hearing loss substantially impacts the quality of life of patients and creates a huge economic burden worldwide^1,2^. The effects of hearing loss are not limited to the sensory domain but also affect the central nervous system, leading to cognitive dysfunction, mood disorders^3,4^, morbidity, and even mortality^5^.

Various therapeutic approaches have been suggested to treat sensorineural hearing loss, including pharmacological agents and gene- and cell-based therapy^6,7^. However, no treatment can regenerate fully functional hair cells, which can transduce mechanical sounds to the central auditory pathway as electric signals. For this reason, devices that can augment or replace hearing, including hearing aids and cochlear implants, are widely used worldwide.

During the past few decades, there have been significant advancements in cochlear implant technology. Such implants have become the treatment of choice for profound hearing loss, regardless of patient age. This technique involves surgical implantation of a device with electrodes within the cochlea to transfer the electric signal to the auditory ganglion.

Electro-acoustic stimulation or hybrid implantation can deliver both acoustic and electric signals to the inner ear^8^, making it suitable for patients with high-frequency hearing loss and intact low-frequency hearing. Preservation of residual low-frequency hearing is essential in order to enhance both acoustic and electric hearing using this device. Various methods have been suggested to protect residual hearing, such as gradual insertion of the electrode, use of a thinner electrode, and application of systemic or topical steroids^9–16^. In terms of drug application for hearing preservation, steroids have anti-inflammatory effects and can be administered via various delivery methods, including osmotic pumps^9–16^. The use of such pumps can modulate the effects of pharmacological agents^9,11,12,16^. The maximal effects on hearing preservation are expected when the optimal concentrations and quantities of pharmacological agents are achieved using osmotic pumps. However, several studies have reported variable outcomes in terms of hearing preservation when using osmotic pump steroid delivery^9,17–19^.

During electrode implantation, cochlear damage may occur due to direct trauma and inflammation. Direct trauma induced by the force of electrode insertion can be minimized by gradual and less-forceful insertion. Inflammation can lead to tissue overgrowth near an electrode. Several new drugs have been introduced to address this^20^, but have failed to show sufficient evidence for clinical use. Furthermore, traditional, natural anti-inflammatory agents have also been used, including sialyllactose (SL), which is a component of human milk and has various anti-inflammatory effects on the body^21^. In the present study, an animal model and dummy electrode to simulate damage due to electrode insertion were used. SL was evaluated in vitro using HEI-OC1 cells and delivered by osmotic pump to protect residual hearing in this animal model. Based on functional and histological outcomes, SL effectively protected residual hearing.

## Results

### HEI-OC1 cell viability

**Figure 1:**
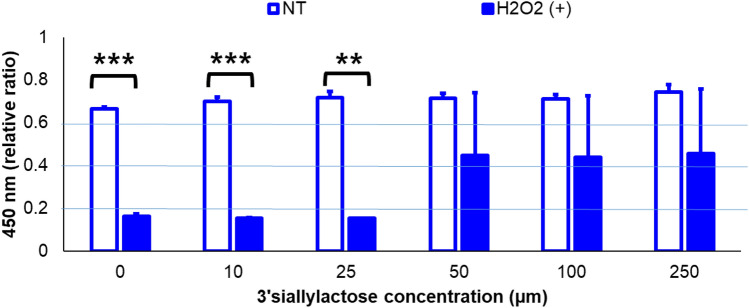
Outcomes of CCK-8 assays of HEI-OC1 cells. Cell viability was analyzed using CCK-8 assays. HEI-OC1 cells were treated with H_2_O_2_ and different concentrations of SL. Viability differences between the control and H_2_O_2_ groups under varying concentrations of SL were compared. The difference in cell viability between the control and H_2_O_2_ groups was larger at low concentrations of SL. However, as the concentration increased, viability decreased. There were significant differences between the control and H_2_O_2_ groups at three lower concentrations of SL (0, 10, and 25 µL). Meanwhile, there were no significant differences at the three higher concentrations (50, 100, and 250 µL). *p < 0.05, **p < 0.01, and ***p < 0.001. NT: no treatment.

The viability of HEI-OC1 cells, which are representative cell lines for inner are hair cells, treated with H_2_O_2_, to mimic the oxidative stress that could be resulted from cochlear implant trauma, and different concentrations of SL (0, 10, 25, 50, 100, and 250 µL) was analyzed (experimental schedule illustrated in Fig. 1). The average cell viabilities of control were around 0.6–0.7 (relative ratio) at all concentrations of SL, whereas those of the H_2_O_2_ groups gradually increased with an increase in the SL concentration (relative ratio = 0.1–0.5). The values differed between the control and H_2_O_2_ plus SL groups, the difference being greater (and significant) at lower concentrations of SL; there were no significant differences at the three higher concentrations (50, 100, and 250 µL) (Fig. 1). These results indicate that SL positively affected the viability of HEI-OC1 cells against H_2_O_2_ toxicity.

### ABR threshold

**Figure 2:**
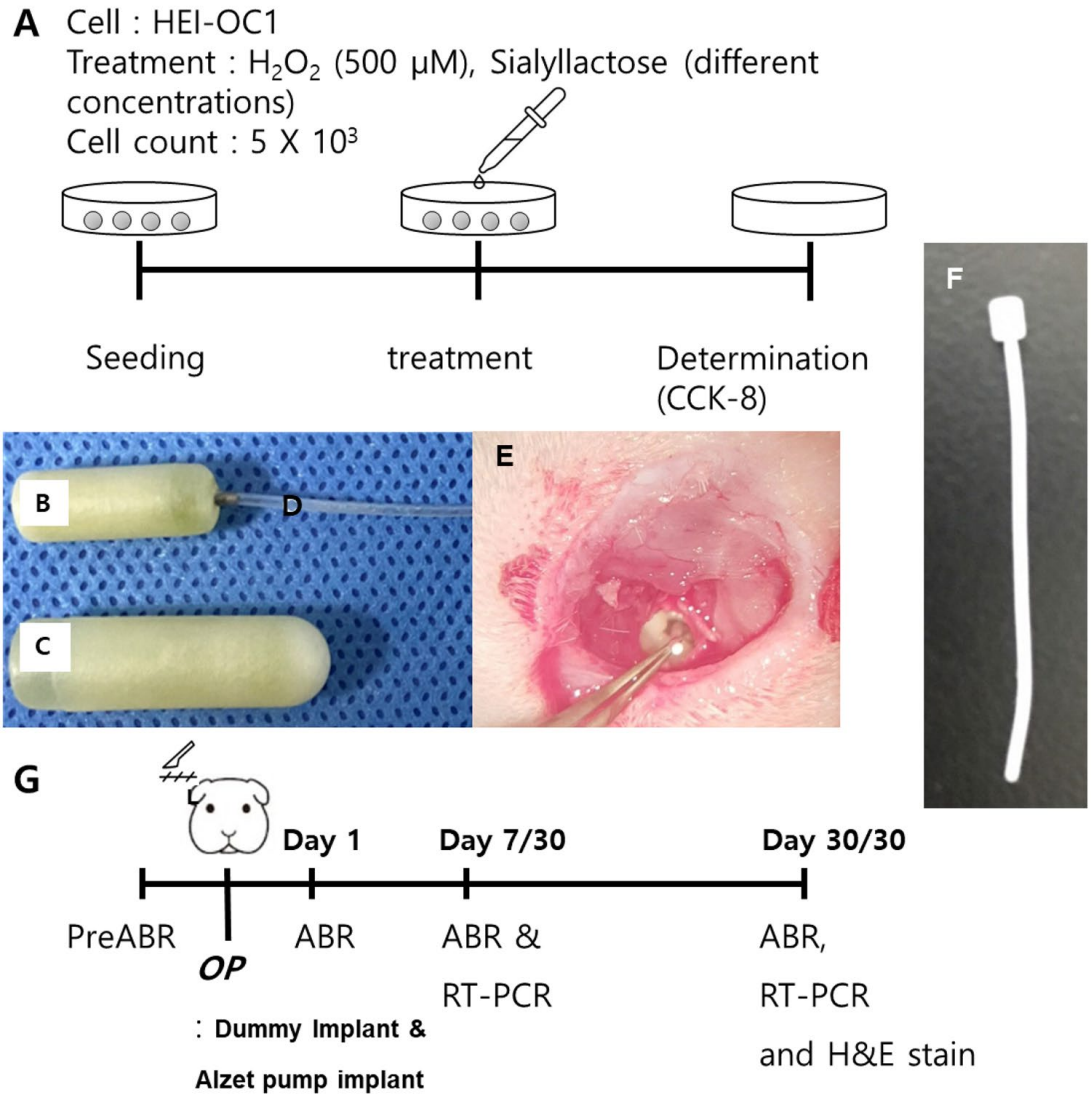
Experimental equipment and study design. (**A**) For the in vitro study, an identical number of HEI-OC1 cells was cultured with H_2_O_2_ and different concentrations of SL. After 24 h, cell viability was assessed using CCK-8 assays. For drug delivery, osmotic pumps with different capacities (1004 and 2004 pumps) (**B** and **C**) were used. The pumps were connected to a microcatheter (**D**) to deliver the pharmacological agent to the target tissue. Photograph of surgical procedures (**E**) showing the implanted dummy electrode through the round window with exposure of the cochlea by removing the bony wall of bullae. A schematic of the study design is presented at the bottom. (**F**) A image of ‘dummy implant’ is shown. (**G**) Auditory brainstem responses were measured four times to determine the threshold: preoperatively and 1, 7, and 30 days postoperatively. RT-PCR was performed at 7 and 30 days after the operation. The histological analysis was performed 30 days after the operation.

To evaluate the hearing ability of experimental animals, the ABR thresholds at 8, 16, and 32 kHz were measured before dummy electrode insertion (baseline) and 1, 7, and 30 days after the surgery (Fig. 2). The threshold shifts, i.e., differences in the threshold compared to baseline, were also calculated.

Osmotic pumps (models 1004 and 2004; Alzet Osmotic Pump, Alza Corp., Palo Alto, CA, USA) used to deliver the SL were implanted simultaneously during dummy electrode insertion. The low-capacity pump (SL-1004) delivered 100 µL SL for 4 weeks (0.11 µL/h), whereas the high-capacity pump (SL-2004) delivered 200 µL SL for 4 weeks (0.25 µL/h). The experimental animals were randomly divided into control (electrode insertion only), SL-1004c [only pump control: low-capacity pump filled with phosphate buffered saline (PBS) + electrode insertion], SL-2004c (only pump control: high-capacity pump filled with PBS + electrode insertion), SL-1004 (low-capacity pump with SL), and SL-2004 (high-capacity pump with SL) groups. The experimental schedules are presented in Fig. 2. There were statistical differences among five groups at all frequencies in all time points.

**Figure 3:**
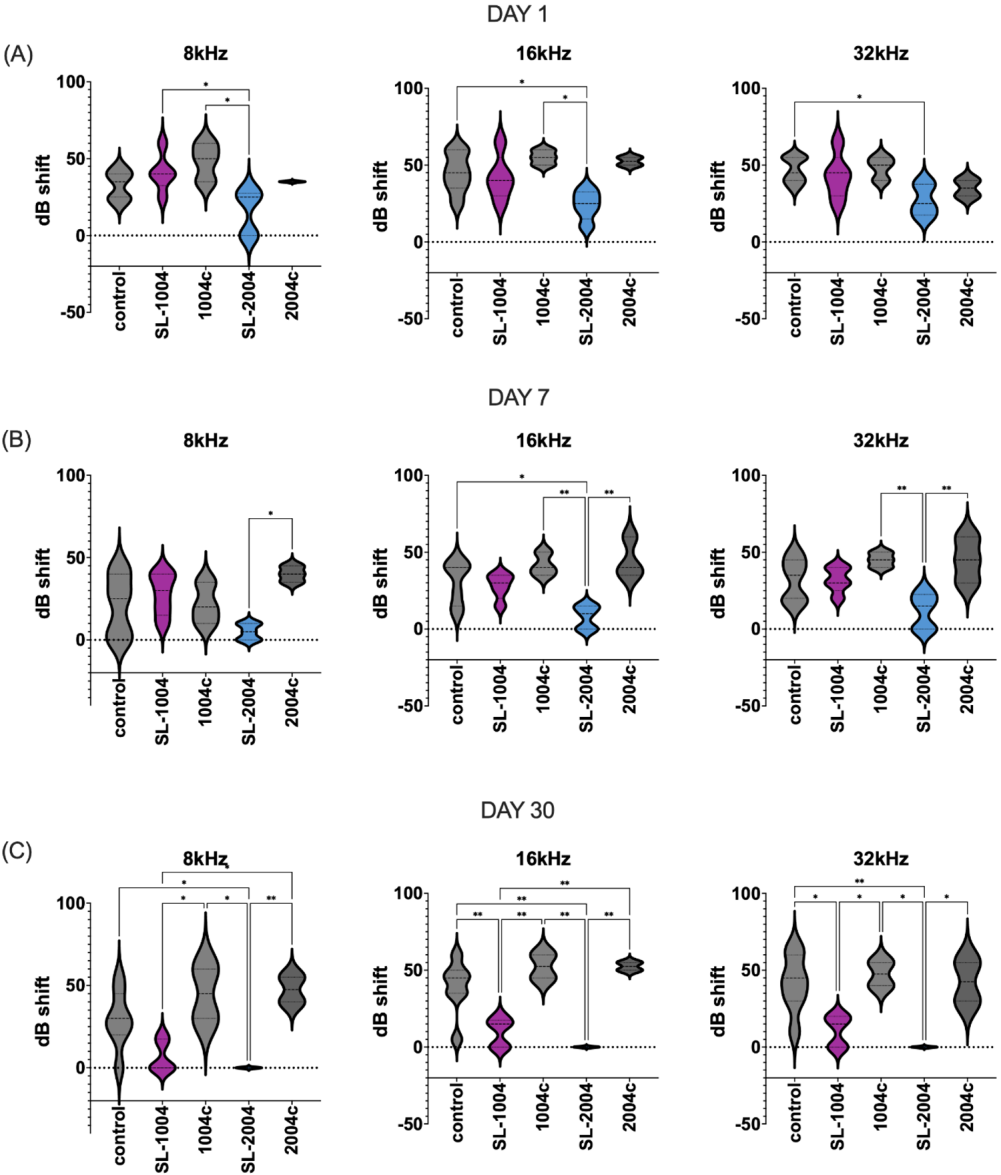
ABR threshold shift of three groups at three time points. The ABRs were measured at 8, 16, and 32 kHz. The experimental groups included no pump control (dummy electrode insertion only), control with pumps (dummy electrode and 1004 or 2004 pumps filled with PBS), and two groups of SL treatment (SL-1004: low-capacity pump; SL-2004: high-capacity pump). The threshold shifts were compared to the baseline value (preABR) and averaged. (**A**) At 1 day after dummy electrode insertion, the difference in threshold shifts at all frequencies among the groups was evaluated. Significant differences in the shift between the control and SL-2004 groups compared to control were found at 16 or 32 kHz. (**B**) At 7 days after dummy electrode insertion, differences among groups were found at all frequencies. At 16 and 32 kHz, significant differences were observed between the 2004c and SL-2004 groups. (**C**) At 30 days after dummy electrode insertion, a difference in threshold shift among the groups was found at all frequencies. At all frequencies, both control with pump groups (1004c and 2004c) showed a significantly higher threshold shift compared to the two SL groups. SL-2004 group showed statistically smaller threshold shift compared to control at all frequencies. *p < 0.05, **p < 0.01, and ***p < 0.001.

One day after surgery, differences in the ABR threshold shifts among five groups were statistically significant only at all frequencies. The threshold shift of the SL-2004 group was significantly lower than that of controls at 16 and 32 kHz, significantly lower than that of 1004c at 8 and 16 kHz, and significantly lower than that of SL-1004 at 8 kHz. At 7 days after surgery, there was a persistent ABR threshold shift difference in SL-2004 group. The differences in ABR threshold shifts were statistically smaller significantly compared to 1004c and 2004c at 16 and 32 kHz. In addition, ABR threshold shift was statistically smaller in SL-2004 compared to 2004c and control at 8 and 16 kHz respectively. At 30 days, the differences were significant across all frequencies (Fig. 3). In both pumps (1004 and 2004), SL causes statistically smaller threshold shifts at all frequencies. Furthermore, at 16 and 32 kHz, both SL groups (SL-1004 and SL-2004) showed statistically smaller threshold shift compared to control. At 8 kHz, SL-2004 group showed statistically smaller threshold shift compared to control. These results demonstrate that continuous delivery of both pharmacological agents prevented the hearing deterioration induced by electrode insertion. Early protective effects (1 day) were demonstrated in the high concentration SL group. At the later stage (30 days after surgery), SL delivery with both types of osmotic pump showed significantly improved ABR thresholds.

### Histological analysis

**Figure 4:**
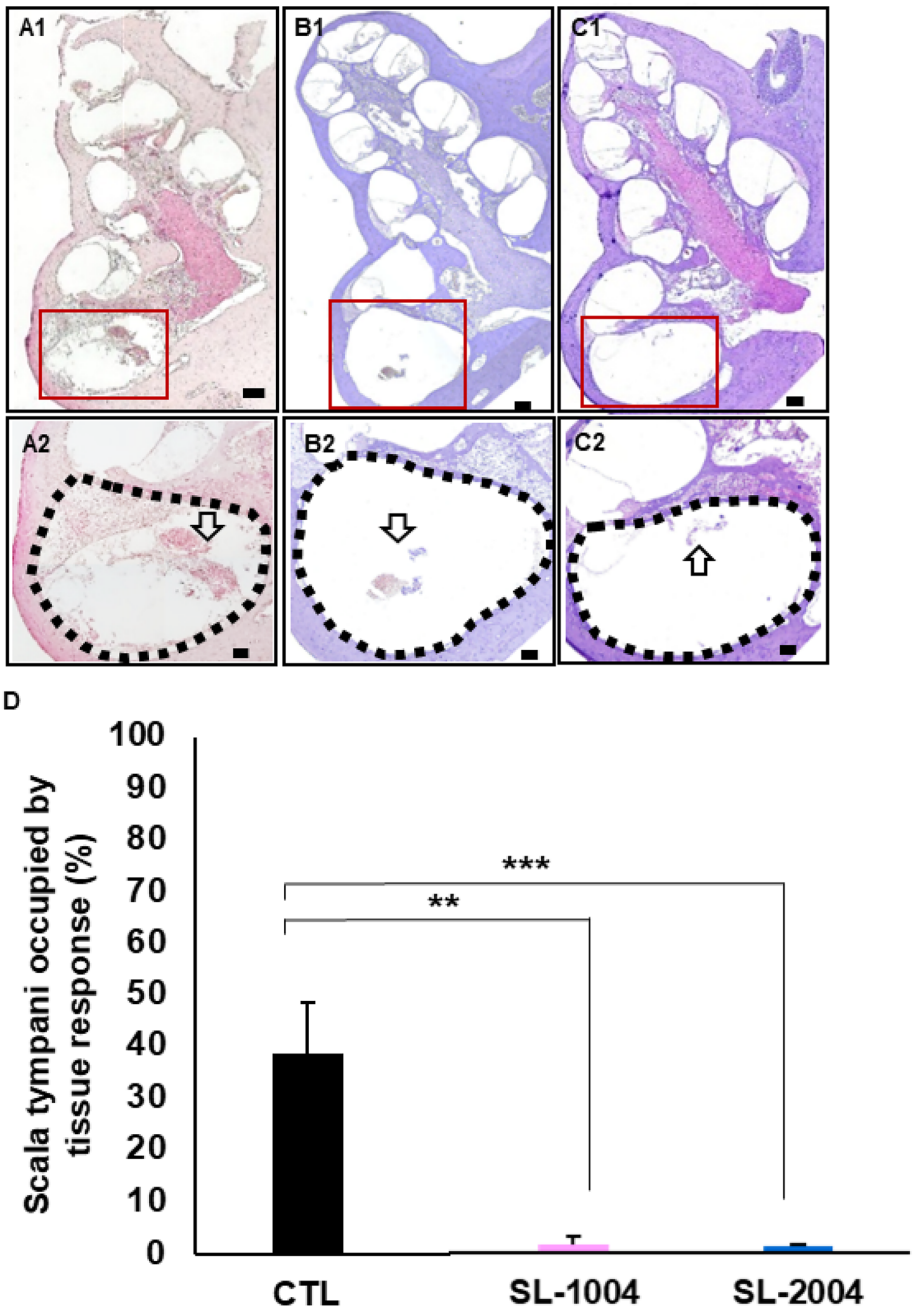
Representative images of H&E staining and quantitative analysis of the extent of fibrosis. Cochleae were harvested at day 30 and prepared for histological analysis (H&E stain). Sectioned cochleae at low magnification (**A**–C1) and high magnification (**A**–C2) are shown. (**A**–**C**) indicate the control, SL-1004, and SL-2004 groups, respectively. The scala tympani with the inserted electrodes is indicated by the dotted line. Tissue responses, such as fibrosis and ossification, were observed within the scala tympani cavities and averaged (**D**). There were significant differences in the tissue responses among groups. Compared to the control group, significantly weaker responses were observed in the SL-1004 and SL-2004 groups. **p < 0.01 and ***p < 0.001. Error bars indicate standard deviations. Scale bars were 200 µm at low magnifications and 100 µm at high magnifications.

Experimental animals were sacrificed on the last day of ABR, and their cochleae were harvested. The sampled cochleae were prepared for sectioning and H&E staining. Images of stained cochleae are shown in supplementary Figs. 1–3 and Fig. 4. After dummy electrode implantation, morphologic changes such as hydrops of Reissner’s membrane occurs in cochlea. The loss of hair cells but intact supporting cells were observed (Supplementary Fig. 1). In comparison among groups, no difference of spiral ganglion morphology was observed among control, SL-1004 and SL-2004 group (Supplementary Fig. 2). Compared to the control group, a smaller degree of soft tissue ossification and fibrosis was observed in both SL groups. The responses within the scala tympani significantly differed among the groups, with significantly weaker responses in both SL groups (Fig. 4). In more apical regions, subtle fibrotic tissue was observed in control group but none in SL groups (Supplementary Fig. 3). These results indicate a good correlation among the histological and functional data in the SL treatment groups, with both sets of data showing favorable outcomes.

### RT-PCR

**Figure 5:**
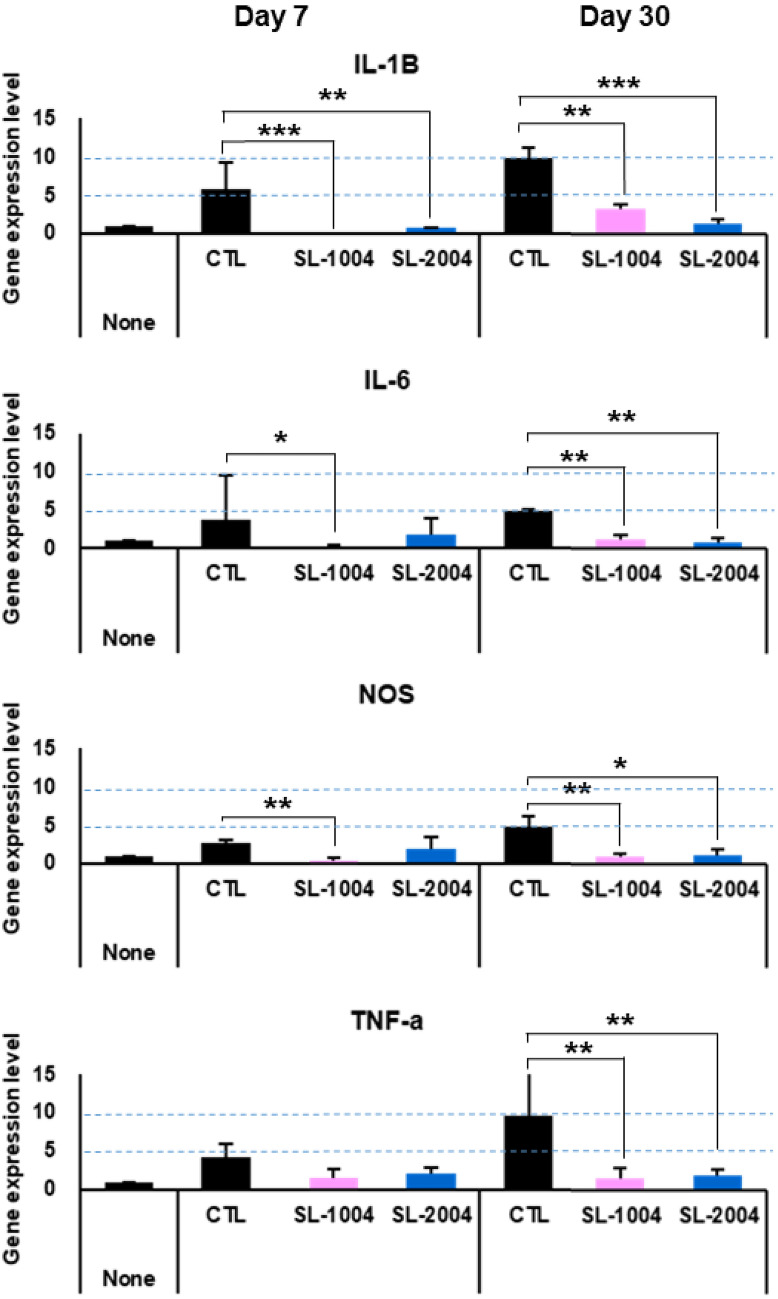
Quantitative RT-PCR of inflammatory cytokines at 7 and 30 days after dummy electrode insertion. The gene expression levels of four cytokines (IL-1B, IL-6, NOS, and TNF-α) were compared among the groups at 7 days (left column) and 30 days (right column) after dummy electrode insertion. At 7 days, the experimental groups had significantly lower expression levels of IL-1B (SL-1004 and SL-2004), IL-6 (SL-1004), and NOS (SL-1004) compared to the control group. At 30 days, all were significantly lower in both SL groups. *p < 0.05, **p < 0.01, and ***p < 0.001. Error bars indicate standard deviations.

To investigate the causes of our findings, the expressions of several inflammatory genes were quantified via RT-PCR (IL-1B, IL-6, NOS, and TNF-α). At first week, the gene expressions of all inflammatory markers, except TNF-α, were significantly different among the groups; they were generally lower in the SL-1004 group. However, the expression of IL-1B was lower in the SL-2004 group than in the SL-1004 group. At 30 days, the gene expressions of all inflammatory markers were significantly different among the groups. Both SL groups had significantly lower expressions of IL-2B, IL-6, and TNF-α than the control group (Fig. 5).

SL-1004 induced rapid (early) changes in gene expression. The variable expression at 1 week suggests that the pharmacological effects varied across groups at early stage. Inflammatory genes (IL-6 and TNF-α) were quantified in both PBS pumps (1004 and 2004). At 1 week, both PBS pumps showed similar level with control (only dummy electrode without any pump): 4.76529- and 6.88932-fold increase of IL-6/11.01356- and 11.06746-fold increase of TNF-α compared to normal respectively. At 2 week, low capacity PBS pump showed 4.98629-fold increase of IL-6/12.18317-fold increase of TNF-α which is similar level with control (only dummy electrode without any pump). The high capacity pump showed rather increase level of inflammatory markers at 30 days. It showed 12.49951-fold increase of IL-6/31.84212-fold increase of TNF-α compared to normal.

## Discussion

Hearing deterioration and tissue reactions, such as fibrosis and ossification, after dummy electrode insertion were observed. In addition, increased gene expressions of inflammatory cytokines were identified in the experimental group. SL administration reduced the hearing threshold shifts following dummy electrode insertion, tissue reactions, and expressions of inflammatory markers. These results could help guide the development of new pharmacological agents for hearing preservation and aid the determination of the underlying mechanism of hearing loss after cochlear implantation.

In the histologic observation of this study, morphological changes of organ of Corti near the dummy electrode insertion were observed (Supplementary Fig. 1). In this image we have revealed that cochlear hair cells were damaged remaining the structure of supporting cells. Further nervous structures were intact at 1 month after dummy electrode insertion. This result implicates that hair cells not supporting cell is damaged after trauma and post traumatic change. In the prior publication about human histologic analysis of cochleae after cochlear implantations which had extensive trauma due to extended round window approach, similar preservation of supporting cells and correlating neuronal structures were observed^22^. These results suggest the possibility of isolated damage of cochlear inner and outer hair cells after electrode insertion into scala tympani.

In the present study, osmotic pumps were used, which can deliver pharmacological agents with varying concentrations and durations of action. The inflammation due to electrode positioning within the scala tympani of cochlea worsens over time^23^. Therefore, drug delivery during cochlear implantation would only affect the initiation of the inflammatory reaction and would have minimal effects on its maintenance in later stages. Conversely, the use of this pump enabled prolonged delivery of pharmacological agents. It could be suggested that, along with the drug-eluting electrodes^24–28^, which release the drug gradually after surgery, this pump has favorable effects on hearing preservation. Our results support previous studies that have reported the beneficial effects of using steroids and osmotic pumps on hearing preservation^9,11,12,16^. The clinical applicability of osmotic pumps requires careful consideration as cochlear implants are popular worldwide, and there are increasing demands for expanding their use. Considering the current procedures, the clinical application of these pumps appears to be useful. Osmotic pumps can be implanted in the soft tissue pocket near the skin incision away from the inner part of the cochlear implant. But to confirm the safety of these pumps in clinical condition, further study is necessary.

Several studies have demonstrated the favorable outcomes of using steroid osmotic pumps. However, few experiments have been performed, the detailed outcomes have varied^9,11,12,16^. In addition, the effects on hearing preservation are not robust, and the underlying mechanisms are still unknown. Conversely, in the present study, the use of anti-inflammatory pharmacological agents has demonstrated robust preservation of the hearing threshold and corresponding histological outcomes. This is in line with the decreased gene expressions of inflammatory markers, which are increased during cochlear implantation^29–32^, suggesting a favorable outcome with regard to protection against electrode insertion-related hearing loss compared to steroid use. Notably, a hearing threshold shift was found in our study. At 1 day after the surgery, the SL-2004 group had lower threshold shifts than the other groups at all frequencies. Moreover, there was a significantly lower threshold shift in the SL-2004 group than in controls. Considering the short time after the surgery, this outcome might have been due to the relatively less-invasive surgical technique or variation in the surgical process, such as anatomical variation and surgeon preference, rather than pharmacological interaction. However, these findings do not negate the evidence of the positive effects of pharmacologic agents used for hearing preservation, as the remaining parameters, including histological and molecular analyses, were improved compared to controls. In addition, except for the SL-2004 group, all groups showed similar threshold shifts at the first day, indicating that the surgical technique has low variability among the other groups.

An osmotic pump can be used clinically during cochlear implantation once several concerns have been addressed. Meanwhile, the side effects of the pharmacological agents should be evaluated further. Off-target effects should be evaluated before the clinical application. For example, via passages such as cochlear aqueduct, central migration of such pharmacologic agent could induce unwanted changes in neural structure. Most of all, one of the underestimated factors of current study theory is the impact of intracochlear electrical stimulation. The current study used dummy electrodes without actual electric stimulation function. There are some evidence that electric stimulation can alter the soft tissue in the body^33,34^. Therefore, there is possibility that these electrical effects are unexpectedly contributing to the alteration of fibrosis between neural structure and cochlear implant electrode in actual clinical circumstances. In-depth studies regarding electric stimulation and cochlear implant fibrosis should be performed to relieve these worries.

## Materials and methods

### HEI-OC1 cell maintenance and viability

HEI-OC1 cells (purchased from SigmaAldrich®; Merck: https://www.sigmaaldrich.com) were cultured in high-glucose DMEM supplemented with 10% (v/v) fetal bovine serum and maintained at 33 °C in a humidified incubator under 10% CO_2_ in air. After culturing HEI-OC1 cells (5000 cells/well) for 24 h, cell viability was assessed using a Cell Counting Kit-8 (Dojindo Laboratories Co., Ltd., Kumamoto, Japan) following treatment with H_2_O_2_ (500 μM) and varying concentrations (0, 10, 25, 50, 100, 250, and 500 µL) of SL for 24 h, in accordance with the manufacturer’s instructions.

### Experimental schedule and animals

Five-week-old male albino guinea pigs were used. The animals were maintained at a controlled temperature (24 °C) and humidity (40–45%), with a 12 h light/dark cycle to simulate the circadian rhythm. The study protocol was approved by Ajou University Medical Center—Institutional Animal Care and Use Committee (protocol No. 2018– 0005). All experiments were performed in accordance with relevant guidelines and regulations and the study is reported in accordance with ARRIVE guidelines. SL (Biosynth® Ltd—Compton, United Kingdom) was used as the pharmacological agent. Total of 23 animals were included. Seven animals for control group, 3 animals for each control pump groups with no pharmacologic agent, and 5 animals for each SL pump groups.

### Auditory brainstem response (ABR)

ABR thresholds were measured by determining the lowest stimulus intensity that provoked a V wave in the ABR response. Animals were anesthetized via intramuscular injection of 0.1 cc/100 g Zoletil (Virbac Corporation France SAS, Virbac Laboratories, Darros, France) and 0.02 cc/100 g 2% Rompun (Bayer Corp., Leverkusen, Germany). The ABR was measured in a soundproof chamber using specialized equipment (Tucker-Davis Technologies Inc, Gainesville, FL, USA) and Biosig32 system software. Three electrodes were positioned at the vertex (+), ipsilateral retroauricular area (−), and contralateral retroauricular area (ground). Tone bursts at three frequencies (8, 16, and 32 kHz), a repetition rate of 11.1/s, and 512 sweeps were used. ABRs were measured before insertion and 1, 7, and 30 days after insertion.

### Dummy electrode insertion

Animals were anesthetized as described previously. Using an aseptic technique, a 1.5 cm retroauricular incision was made. Bullae were identified under a microscope, and the lateral walls of the bullae were removed. Then the cochlear round window was visualized. Next, a small incision was made on its membrane, and a dummy electrode composed of silastic components (Daegu Gyeongbuk Institute of Science and Technology, Daegu, Korea; shaft diameter, 0.64 mm; tip diameter, 0.43 mm) was gradually inserted into the scala tympani of the cochlea (Fig. 2). The electrode was inserted until resistance was met, thus the depth of insertion is not exact but about 5 mm in average, which corresponds to the 24–32 kHz frequency region. This specific surgical procedure is reproducible in between and different studies.^9,11,12,16^ The osmotic pump was implanted in the retroauricular subcutaneous pocket and connecting microcatheter opening was placed on the round window adjacent to the implanted dummy electrode. This microcatheter placement did not alter the surgical technique for round window exposure.

### Reverse transcription polymerase chain reaction (RT-PCR) and histology

At 7 and 30 days following dummy electrode insertion, three animals per each group were sacrificed for RT-PCR followed by removal of their cochleae. RNA was extracted using RNAiso Plus (9108; TaKaRa Inc., Shiga, Japan), and the extracted RNA was homogenized using an RNAiso Plus homogenizer. The supernatant was isolated after centrifugation at 13,000 rpm and 4°C for 5 min. After adding 0.2 mL chloroform, the supernatant was isolated via centrifugation under the previously described conditions for 15 min. Then isopropyl alcohol was added, and the solution was allowed to rest for 10 min. After another round of centrifugation under the same conditions for 10 min, the supernatant was discarded, and the remaining solution was diluted using DEPC-DW. Then cDNA was conjugated using a Primescript 1 strand cDNA synthesis kit (6110A; TaKaRa Inc., Shiga, Japan). In total, 10 µL Oligo dT primer (50 µmol, 1 µL), dNTP mixture (10 mL, 1 µL), template RNA (1 µL), and RNaser Free dH_2_O_2_ (7 µL) were mixed. After incubating the mixture at 65°C for 5 min, it was stored on ice. Then it was mixed with 4 µL of 5X Primescript buffer, RNase inhibitor (50 U/µL, 0.5 µL), Primesciprt RTase (200 U/µL, 1 µL), and RNase free dH2P (4.5 µL), under careful stirring. Next, it was gradually heated (30 °C for 10 min, 42 °C for 45 min, and 5°C for 5 min), followed by storage on ice. Using a SYBR green 1 qPCR kit (RR420; TaKaRa Inc., Shiga, Japan) and CFX Connect Real-Time PCR Detection system (Bio-Rad Co., Hercules, CA, USA), RT-PCR was performed to evaluate the gene expressions of IL-1B, IL-6, NOS2, and TNF-α. The primers of these cytokines are listed in Table 1. The Minimum Information for Publication of Quantitative Real-Time PCR Experiments (MIQE) and other detailed information are shown in supplementary Tables 2–4 based on guidelines^35,36^.

**Table 1:** Oligonucleotide primers used for quantitative real-time PCR.

Primer name		Primer	
GAPDH	Forward	5′-GCCCTCAATGACCACTTTGT-3′	
	Reverse	5′-TGCTGTAGCCGAACTCATTG-3′	
IL-1β	Forward	5′-TCCCTGTGAAAACAAGAGCA-3′	
	Reverse	5′-CGCCTTTCTCTTGGAGCTTA-3′	
IL-6	Forward	5′-AATTCCTGAGCCCAACTCCA-3′	
	Reverse	5′-TGCTTTCCGAATAGCCCTCA-3′	
TNF-α	Forward	5′-ATCAAGAGTCCCTGCCAGAA-3′	
	Reverse	5′-CTCCCAGGTAGATGGGTTCA-3′	
NOS2	Forward	5′-CCCTCTTCGTGCTGAAAAAG-3′	
	Reverse	5′-GTCATGAGCAAAGGCACAGA-3′	

Oligonucleotides used for PCR for the expression levels of IL-1β, IL-10, TNF-α, and NOS2.

*IL* interleukin, *TNF* tumor necrosis factor, *NOS2* nitric oxide synthase 2.

After the experiment, animals were sacrificed for histological analysis. Cochleae were harvested and fixed with 4% paraformaldehyde for 72 h and then placed in Calci-Clear Rapid solution (National Diagnostics, Atlanta, GA, USA) for 7  days for decalcification. After decalcification, a paraffin block of the specimen was created, and Sects. 3.5 µm thick were created using a microtome. The sections were stained with hematoxylin and eosin (H&E), and observed under a microscope (BX51; Olympus Co., Tokyo, Japan). The degree of tissue reaction within the scala tympani was quantified using ImageJ software (NIH)^9,16^.

### Statistical analysis

Data are expressed as means ± standard errors of the mean. SPSS software (version 23.0; IBM Corp., Armonk, NY, USA) was used for data analysis. An independent t-test was used to compare two groups. For comparisons of more than two groups, one-way analysis of variance and Tukey’s post hoc test were used. *P*-values < 0.05 were considered indicative of statistical significance.

### Supplementary Information

Supplementary Figures.

Supplementary Tables.

## Data Availability

The datasets used and/or analysed during the current study available from the corresponding author on reasonable request.

## References

[1] Härkönen K, Kivekäs I, Rautiainen M, Kotti V, Vasama JP (2017). Quality of life and hearing eight years after sudden sensorineural hearing loss. Laryngoscope.

[2] Chadha S, Kamenov K, Cieza A (2021). The world report on hearing, 2021. Bull. World Health Organ..

[3] Loughrey DG, Kelly ME, Kelley GA, Brennan S, Lawlor BA (2018). Association of age-related hearing loss with cognitive function, cognitive impairment, and dementia: A systematic review and meta-analysis. JAMA Otolaryngol. Head Neck Surg..

[4] Rutherford BR, Brewster K, Golub JS, Kim AH, Roose SP (2018). Sensation and psychiatry: Linking age-related hearing loss to late-life depression and cognitive decline. Am. J. Psychiatry.

[5] Chou CL, Hsieh TC, Chen JS, Fang TC (2018). Sudden sensorineural hearing loss in hemodialysis patients could be a marker of pathogenic progression in the mortality and atherosclerotic events: A national cohort study. Otol. Neurotol. Off. Publ. Am. Otol. Soc. Am. Neurotol. Soc. Euro. Acad. Otol. Neurotol..

[6] Holt JR, Raphael Y (2020). Introduction to the hearing research special issue on inner ear gene therapy. Hear. Res..

[7] Lee MY, Park YH (2018). Potential of gene and cell therapy for inner ear hair cells. Biomed. Res. Int..

[8] Lenarz T (2009). Electro-acoustic stimulation of the cochlea. Audiol. Neuro-otol..

[9] Lee MY (2015). Continuous topical drug delivery using osmotic pump in animal cochlear implant model: Continuous steroid delivery is effective for hearing preservation. Acta Otolaryngol..

[10] Kuthubutheen J, Smith L, Hwang E, Lin V (2016). Preoperative steroids for hearing preservation cochlear implantation: A review. Cochlear Implants Int..

[11] Rah YC (2016). Extended use of systemic steroid is beneficial in preserving hearing in guinea pigs after cochlear implant. Acta oto-laryngol..

[12] Chang MY (2017). The effect of systemic steroid on hearing preservation after cochlear implantation via round window approach: A guinea pig model. Otol. Neurotol. Off. Publ. Am. Otol. Soc. Am. Neurotol. Soc. Euro. Acad. Otol. Neurotol..

[13] Skarżyńska MB (2018). Preservation of hearing following cochlear implantation using different steroid therapy regimens: A prospective clinical study. Med. Sci. Monit. Int. Med. J. Exp. Clin. Res..

[14] Shaul C (2019). Glucocorticoid for hearing preservation after cochlear implantation: a systemic review and meta-analysis of animal studies. Otol. Neurotol. Off. Publ. Am. Otol. Soc. Am. Neurotol. Soc. Euro. Acad. Otol. Neurotol..

[15] Gotamco GL (2020). Comparison of hearing preservation outcomes using extended versus single-dose steroid therapy in cochlear implantation. Otol. Neurotol. Off. Publ. Am. Otol. Soc. Am. Neurotol. Soc. Euro. Acad. Otol. Neurotol..

[16] Lee MY (2020). Dexamethasone delivery for hearing preservation in animal cochlear implant model: Continuity, long-term release, and fast release rate. Acta oto-laryngol..

[17] Himeno C (2002). Intra-cochlear administration of dexamethasone attenuates aminoglycoside ototoxicity in the guinea pig. Hear. Res..

[18] Takemura K (2004). Direct inner ear infusion of dexamethasone attenuates noise-induced trauma in guinea pig. Hear. Res..

[19] Eshraghi AA (2007). Local dexamethasone therapy conserves hearing in an animal model of electrode insertion trauma-induced hearing loss. Otol. Neurotol..

[20] Shikata C, Nemoto M, Ebisawa T, Nishiyama A, Takeda N (2011). Effect of sarpogrelate on cardiovascular disorders. Exp. Clin. Cardiol..

[21] ten Bruggencate SJ, Bovee-Oudenhoven IM, Feitsma AL, van Hoffen E, Schoterman MH (2014). Functional role and mechanisms of sialyllactose and other sialylated milk oligosaccharides. Nutr. Rev..

[22] deTorres A (2019). Supporting cell survival after cochlear implant surgery. Laryngoscope.

[23] Smouha EE (2003). Surgery of the inner ear with hearing preservation: Serial histological changes. Laryngoscope.

[24] Farhadi M (2013). Dexamethasone eluting cochlear implant: Histological study in animal model. Cochlear Implants Int..

[25] Kikkawa YS (2014). Growth factor-eluting cochlear implant electrode: Impact on residual auditory function, insertional trauma, and fibrosis. J. Trans. Med..

[26] Astolfi L (2016). Cochlear implant and inflammation reaction: Safety study of a new steroid-eluting electrode. Hear. Res..

[27] Jang J (2019). A 3D microscaffold cochlear electrode array for steroid elution. Adv. Healthc. Mater..

[28] Briggs R (2020). Comparison of electrode impedance measures between a dexamethasone-eluting and standard Cochlear™ Contour Advance® electrode in adult cochlear implant recipients. Hear. Res..

[29] Guzik TJ, Korbut R, Adamek-Guzik T (2003). Nitric oxide and superoxide in inflammation and immune regulation. J. Physiol. Pharmacol. Off. J. Pol. Physiol. Soc..

[30] Warnecke A (2019). Defining the inflammatory microenvironment in the human cochlea by perilymph analysis: Toward liquid biopsy of the cochlea. Front. Neurol..

[31] Maeda K, Yoshida K, Ichimiya I, Suzuki M (2005). Dexamethasone inhibits tumor necrosis factor-alpha-induced cytokine secretion from spiral ligament fibrocytes. Hear. Res..

[32] Yoshida K, Ichimiya I, Suzuki M, Mogi G (1999). Effect of proinflammatory cytokines on cultured spiral ligament fibrocytes. Hear. Res..

[33] Honda Y (2021). Effect of belt electrode-skeletal muscle electrical stimulation on immobilization-induced muscle fibrosis. PloS one.

[34] Luo R, Dai J, Zhang J, Li Z (2021). Accelerated skin wound healing by electrical stimulation. Adv. Healthc. Mater..

[35] Bustin SA (2009). The MIQE guidelines: Minimum information for publication of quantitative real-time PCR experiments. Clin. Chem..

[36] Bustin SA (2010). MIQE précis: Practical implementation of minimum standard guidelines for fluorescence-based quantitative real-time PCR experiments. BMC Mol. Biol..

