# Supplementary material for: Sialyllactose preserves residual hearing after cochlear implantation
Source: Sci Rep. 2024 Jun 10;14:13376. doi: 10.1038/s41598-024-62344-0 (PMC11167013; doi:10.1038/s41598-024-62344-0)

**Supplementary Figure 1. Representative image (H&E stain) of cochlea basal turn after dummy electrode implantation with osmotic pump (PBS)**

A: low magnification; B: high magnification; Black arrow: spiral ganglion; Empty black arrow: peripheral auditory nerve fiber; Black arrow head: residual fibrous tissue; Red arrow head: Reissner’s membrane; Empty red arrow: loss of inner hair cell; Empty blue arrow: loss of outer hair cell; Empty green arrow: intact supporting cells; Scale bars were 200 µm at low magnifications

**
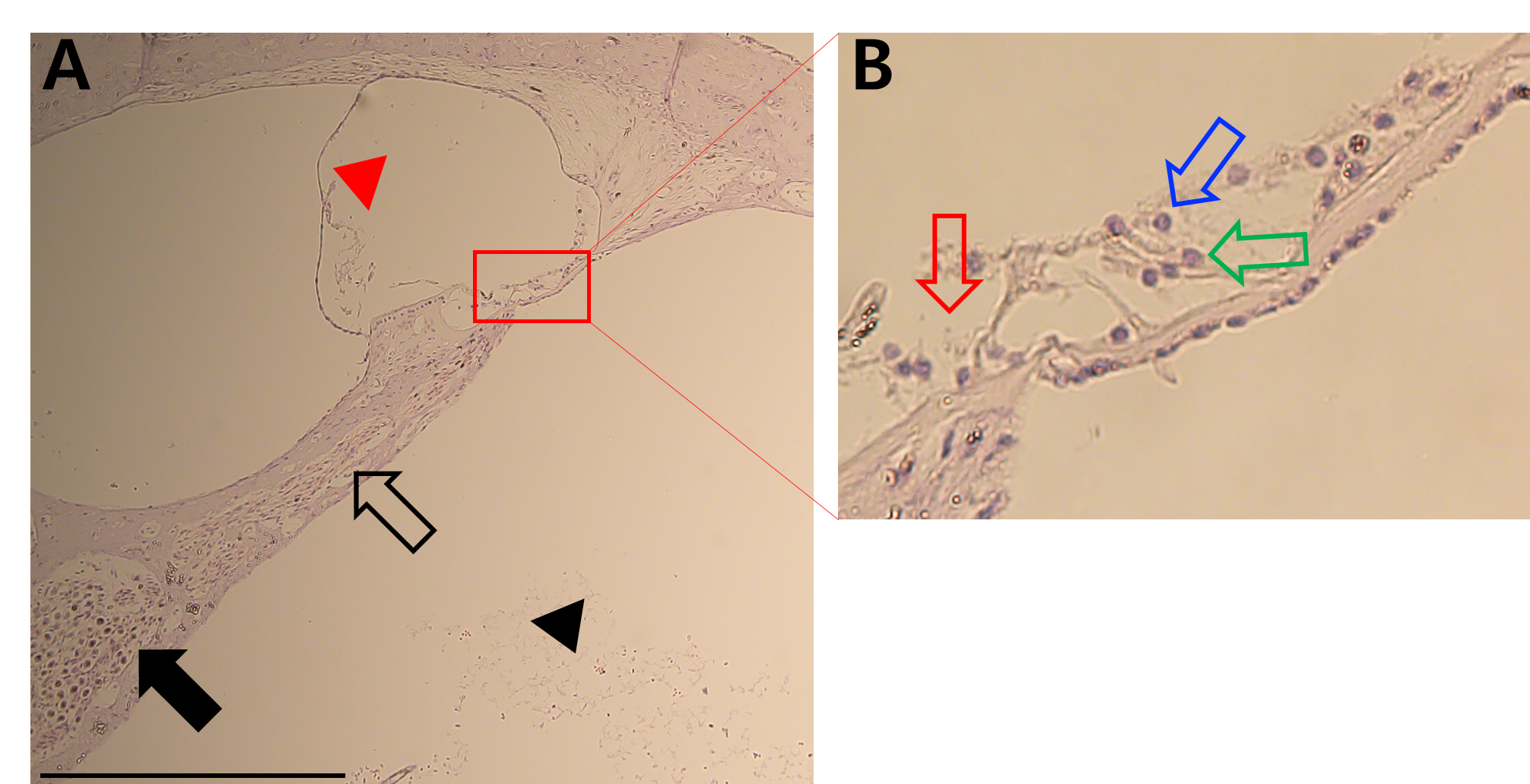
**

**Supplementary Figure 2. H&E stain of middle and basal turns of cochlea (Scale bars were 200 µm at low magnifications)**


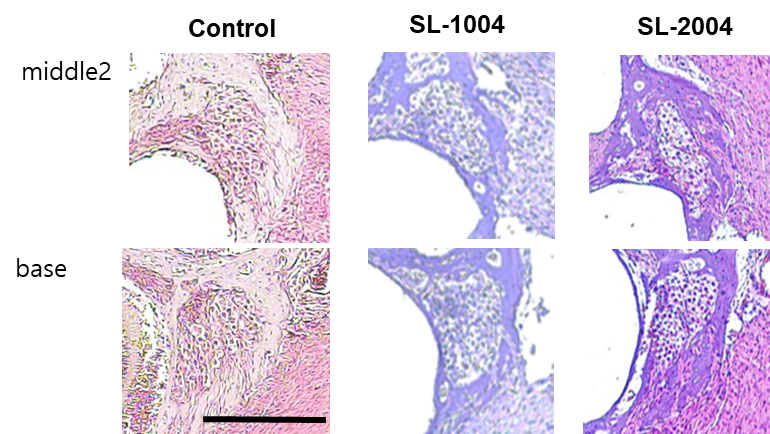


**Supplementary Figure 3. H&E stain of apical and middle turns of cochlea (Scale bars were 200 µm at low magnifications, red dotted line: scala tympani)**


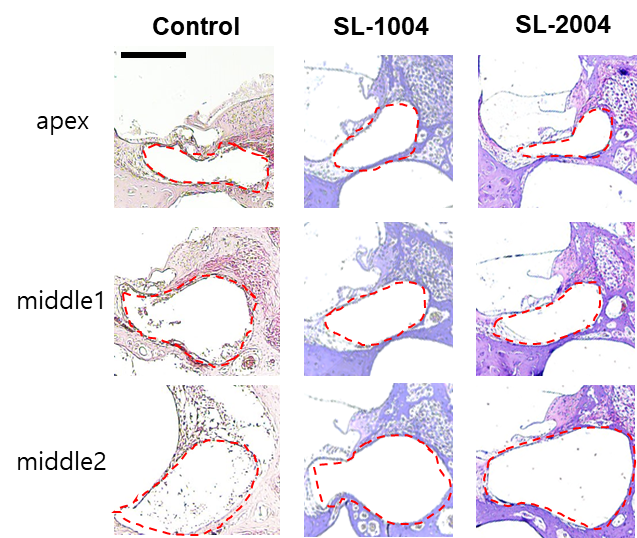

Supplement: Supplementary file 1 — Supplementary Figures. [file 41598_2024_62344_MOESM1_ESM.docx]
